# Skeleton-Based Human Pose Recognition Using Channel State Information: A Survey

**DOI:** 10.3390/s22228738

**Published:** 2022-11-11

**Authors:** Zhengjie Wang, Mingjing Ma, Xiaoxue Feng, Xue Li, Fei Liu, Yinjing Guo, Da Chen

**Affiliations:** College of Electronic and Information Engineering, Shandong University of Science and Technology, Qingdao 266590, China

**Keywords:** Wi-Fi sensing, channel state information (CSI), human pose reconstruction, human skeleton, neural network

## Abstract

With the increasing demand for human-computer interaction and health monitoring, human behavior recognition with device-free patterns has attracted extensive attention. The fluctuations of the Wi-Fi signal caused by human actions in a Wi-Fi coverage area can be used to precisely identify the human skeleton and pose, which effectively overcomes the problems of the traditional solution. Although many promising results have been achieved, no survey summarizes the research progress. This paper aims to comprehensively investigate and analyze the latest applications of human behavior recognition based on channel state information (CSI) and the human skeleton. First, we review the human profile perception and skeleton recognition progress based on wireless perception technologies. Second, we summarize the general framework of precise pose recognition, including signal preprocessing methods, neural network models, and performance results. Then, we classify skeleton model generation methods into three categories and emphasize the crucial difference among these typical applications. Furthermore, we discuss two aspects, such as experimental scenarios and recognition targets. Finally, we conclude the paper by summarizing the issues in typical systems and the main research directions for the future.

## 1. Introduction

With the rapid development of information technology and the increase in people’s health requirements, human behavior recognition has attracted widespread attention [1]. Human behavior recognition is widely used in smart medical care, smart homes, virtual reality, and motion analysis and has strong research importance [2,3,4,5].

Human behavior recognition is divided into device-based [6,7,8] and device-free [9,10,11,12] methods, according to whether the person wears or carries the sensor. Device-based approaches, although generally accurate, are inconvenient in many important real-life scenarios, e.g., requiring the elderly or patients to carry the device at all times. Device-free methods make up for the deficiencies of such scenarios. For device-free human sensing, there is a wide range of sensing technologies available, including microphones and cameras. Some sensors, such as microphones and cameras, raise privacy issues. Radio-frequency (RF) signals offer unique advantages compared to these sensors because they are generally ubiquitous and can protect privacy [13]. In addition, RF signals can be utilized in poor light conditions and traverse obstacles, such as walls.

In the field of noncontact and non-line of sight (NLoS) perception [14], by analogy with data that are collected by sensors, many wireless signals can be used to identify human behavior [15]. Existing RF-based sensing systems use either commodity Wi-Fi devices or dedicated RF devices [16]. For example, radar sensing usually employs specialized devices, and it can be used to extract the target’s location, shape, and motion characteristics [17,18,19,20]. Sensing systems using commercial Wi-Fi hardware can take advantage of Wi-Fi devices to achieve ubiquitous sensing [21]. Early Wi-Fi-based systems used received signal strength (RSS) for coarse-grained sensing [22,23]. However, RSS varies randomly to some extent and does not regularly change with the circumstances. Due to the frequency of diversity [24], signal stability and satisfactory accuracy of CSI, it is broadly used in human behavior recognition [25,26,27,28,29,30,31,32,33].

With the improvement of wireless sensor systems’ algorithms and applications of artificial intelligence [34], a fundamental question arises: Can wireless signals provide the same functionality as cameras for fine-grained human perception, such as human pose recognition? Human pose recognition (HPR) can be understood as the position estimation of the human body’s pose (joints or key points, such as head, left hand and right foot) and has made remarkable achievements in computer vision (CV) [35,36,37]. However, the CV method may cause privacy problems. To address this issue, some researchers apply optical signals to realize human 3D skeleton recognition and achieve satisfactory results [38,39]. In addition, several radio-frequency (RF) sensing methods have been proposed for human pose recognition, such as Wi-Fi [40,41,42,43] and frequency-modulated continuous wave (FMCW) radar [44,45,46,47]. We use wireless sensing schemes to estimate human joints using a confidence map to overcome the technical challenges of traditional CV-based human pose recognition solutions (i.e., occlusion, poor light, clothing, and privacy issues). The map is constructed by RF signals and demonstrates the potential to realize a new generation of applications. In Section 2, we briefly introduce the application of the FMCW radar and Wi-Fi in human pose recognition before comparing their advantages and disadvantages, which provides some useful research directions for initial researchers.

However, in these schemes, radar sensing is not suitable for large-scale deployment due to the high infrastructure cost. Therefore, we are interested in sensing systems utilizing commodity Wi-Fi devices. In a wireless network, the multipath effect is generated during the propagation of wireless signals. Meanwhile, the CSI value changes according to the positions of the human body. Hence, pose recognition can be realized according to the changes in the CSI amplitude and phase. In this work, we mainly discuss the Wi-Fi human pose recognition systems leveraging CSI. Many important works of Wi-Fi CSI human pose recognition have been published in recent years [33,48,49,50,51,52,53,54,55]. In general, a person’s pose is often described by a skeleton [56]. These articles investigate typical application scenarios using CSI to build human skeletons, e.g., single person, multiperson, and 2D and 3D human pose recognition.

There are some surveys on specific types of Wi-Fi CSI sensing. For example, in [57], the survey that has the widest application range, including human detection, motion detection, respiratory monitoring, etc., Wang et al. [58] present a survey on CSI-based behavior recognition applications within six years, and the survey has the widest time. In [59], the authors classify CSI-based applications into localization, macro activity recognition and micro activity recognition. However, there is no comprehensive survey to summarize the crucial technique based on the skeleton and CSI. This survey is different from existing ones in that it has a specific research scope and the most novel research content. Our main contributions are summarized as follows.

1. The first review. We investigate behavior recognition methods that are based on Wi-Fi signals and human skeletons. To the best of our knowledge, this is the first review of human pose recognition using CSI and human skeletons. This paper serves as a practical guide for understanding real-life applications using CSI to identify human skeletons.
2. Comprehensive investigation. We present a general framework of precise pose recognition and comprehensively analyze the system components, which include data processing methods, neural network models, and performance results.
3. Typical skeleton generation model. We classify skeleton model generation methods into three categories: the human silhouette, key point coordinate, and point cloud. We emphasize the crucial difference among these typical applications, as well as the advantages and disadvantages of these models.
4. Discussion and future trends. We extensively discuss related factors that affect pose recognition from six aspects. In particular, we discuss the techniques that may be transferred from computer vision to CSI pose recognition.

**Figure 1:**
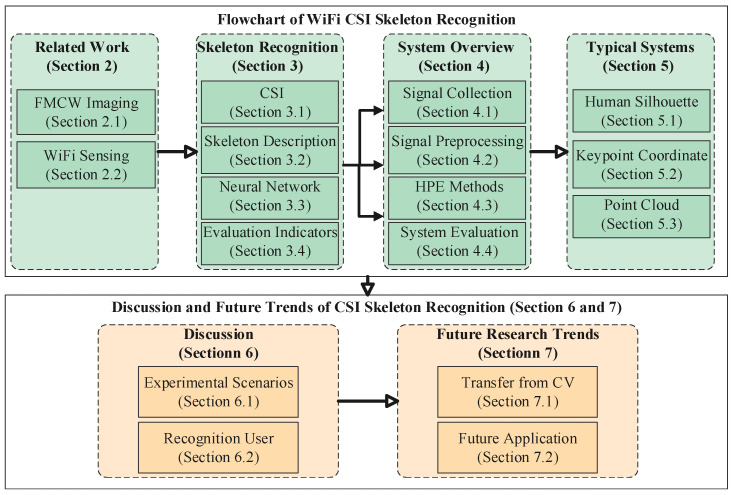
Taxonomy of this survey.

The remainder of this work consists of the following parts, as shown in Figure 1. We make a detailed investigation of Wi-Fi CSI skeleton recognition from two parts. The first part contains related work (Section 2), skeleton recognition (Section 3), system overview (Section 4) and typical systems (Section 6). Related Work on human pose recognition using RF is discussed in Section 2. Section 3 describes the fundamental knowledge of CSI skeleton recognition. Section 4 presents the process of CSI data generation of human skeleton in detail, which include signal collection, signal preprocessing, and pose recognition methods. Section 5 summarizes the typical applications that are based on CSI pose recognition. The second part presents a discussion (Section 6) and future trends (Section 7) of CSI skeleton recognition. Section 6 discusses two aspects: application scenarios and recognition users. Furthermore, the transfer technique from CV and the future application scenarios of CSI skeleton are presented in Section 7. Finally, we conclude our survey in Section 8.

## 2. Related Work on Human Pose Recognition Using RF

In recent years, human pose recognition has attracted widespread attention, and many researchers have realized pose recognition using deep neural network models [60]. For HPR, the reconstruction of the human skeleton is the most important part, which is divided into rough human contours and fine-grained human skeletons. There are two general methods of skeleton generation, namely, regression key point coordinates and body part heatmaps, both of which have yielded remarkable results in CV. However, the CV approach is vulnerable to privacy and light issues.

Recently, smart wireless sensing techniques have achieved promising developments [61]. Based on these successful studies, some authors have proposed human pose recognition schemes that were based on RF sensing, such as frequency modulated continuous wave (FMCW) radar and Wi-Fi, to solve these problems. In the following sections, we introduce the application of FMCW and the Wi-Fi signals in detail in the field of HPR from two representations: rough human silhouettes and fine-grained human skeletons.

### 2.1. FMCW Imaging

Generally, FMCW signals are generated by two types of hardware: a universal software radio peripheral (USRP) software radio that is equipped with an LFRX daughterboard and IWR [62]. FMCW radar systems can achieve high spatial resolution through larger bandwidths and more antennas, enabling fine-grained human perception [63]. In this section, we introduce the applications of pose recognition of human silhouettes and skeletons that are generated by FMCW signals.

#### 2.1.1. Human Silhouette Generation by FMCW

**Figure 2:**
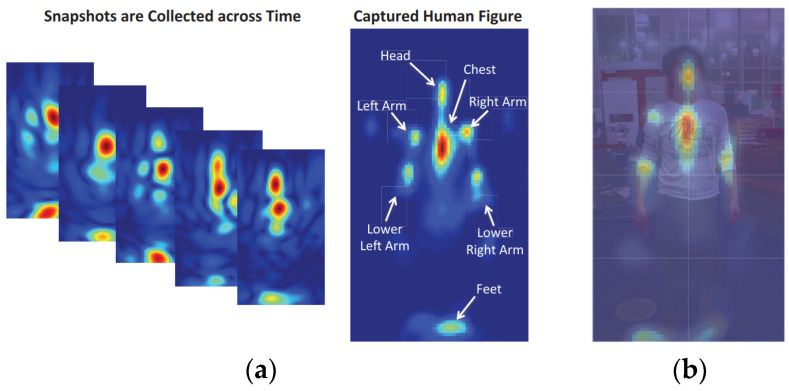
Human Contour was generated by FMCW [44]. Figure (**a**) shows contours of the human body that were generated from snapshots that were collected at various times, and (**b**) compares contours that were generated by RF and the poses of real human bodies.

**Figure 3:**
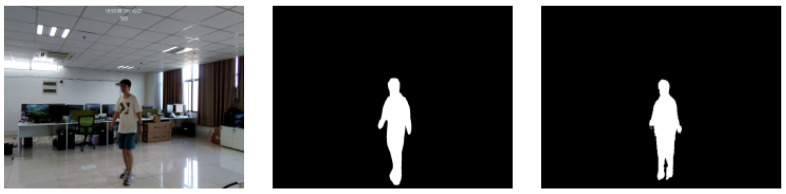
The human profile was generated by FMCW [64]. The images from left to right are the video frames, the ground truth, and the RFMask predicted human silhouette.

Based on FMCW, human sensing systems have tried to generate human silhouettes by RF reflections [44,64]. Specifically, RF-Capture [44] generates a coarse description of the human body behind the wall by collapsing multiple body parts detected at different time points. The system is limited to performing a single-person action, as shown in Figure 2. The RFMask [64] uses millimeter-wave (mmWave) radar to extract accurate human contours to analyze human reflections on the heatmap and create accurate human silhouettes. This system can be extended to achieve multiperson and multi-action human poses in various scenarios (i.e., occlusion, low illumination), which has strong robustness. Figure 3 shows the human silhouette generated by the RFMask without occlusion.

#### 2.1.2. Human Skeleton Generation by FMCW

**Figure 4:**
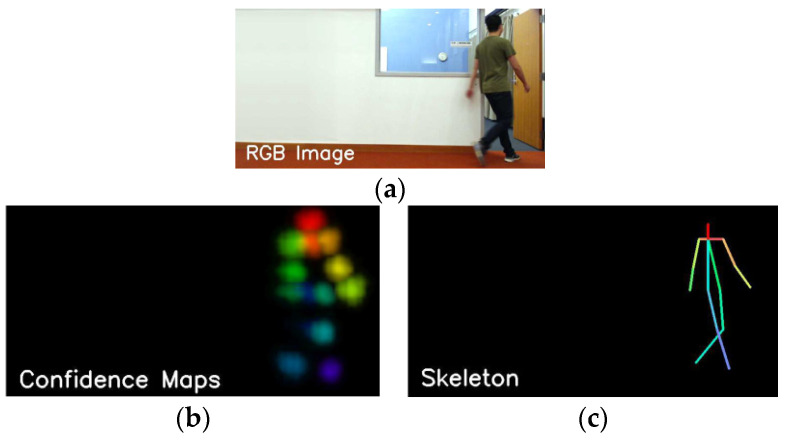
Human Skeletons were generated by FMCW [67]. (**a**) is a ground-truth image that was captured by the camera; (**b**) presents the confidence maps of key points generated by RF signals; (**c**) is the human skeleton that was generated according to (**b**).

**Figure 5:**
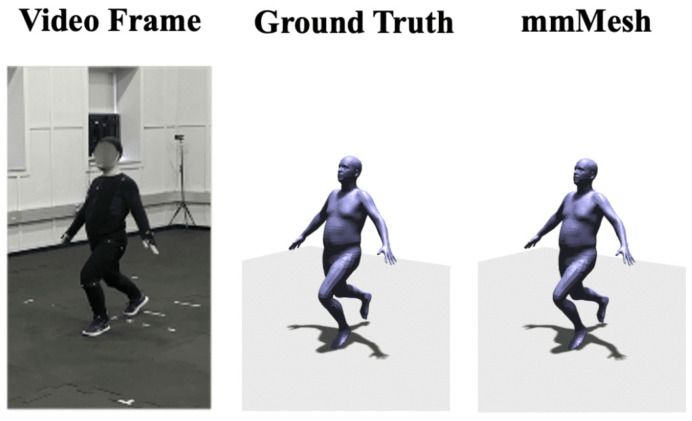
Examples of the constructed 3D human mesh [73]. The images from left to right are the video frames, the ground truth, and the mmMesh predicted human mesh.

At present, wireless signals based on FMCW can generate a fine-grained human skeleton, which forms a complete human shape through the effective connection of human joints or key points [65,66]. For example, RF-Pose [67] and RFPose3D [68] extract accurate 2D and 3D skeletons with occlusion and through a wall. T. Li et al. [69] generated a human skeleton through a wall, which realized the action and interaction of single and multiple people, as shown in Figure 4. In addition, mmWave radar has a much shorter wavelength, enabling high-precision skeleton reconstruction [47,70,71,72]. The mmMesh [73] can dynamically locate the moving subject and capture the person’s body shape and pose. Moreover, it leverages the skinned multi-person linear (SMPL) model to reconstruct a dynamic human mesh in real-time, as shown in Figure 5. The mPose [74] utilizes the CNN regression model to extract 3D skeletal joint coordinates, enabling robust pose reconstruction across users. HPERL [75] is the first study to fuse RGB and LiDAR for 3D human pose recognition. HPERL identifies human contours by analyzing projection and 3D bounding boxes, and then generates 3D predictions using PedX’s 2D keypoint as ground truth. Compared with mmWave radar, LiDAR is rarely used only for human pose recognition because it is expensive and more suitable for long-distance detection.

Compared with human silhouettes, fine-grained skeletons are more robust in the face of complex environments. The human contour graph was generated via a heatmap, and the skeleton was predicted via regression of key points. The skeleton models look more realistic, which can directly demonstrate the behavior of the human body, and it is unaffected by occlusion or light.

### 2.2. Wi-Fi Sensing

With the high demand for wireless data traffic, Wi-Fi networks have rapidly grown because they provide high throughput and are easy to deploy [57]. Due to its lower cost and higher power efficiency, Wi-Fi CSI set off a research surge and yielded research results in pose recognition.

#### 2.2.1. Wi-Fi CSI Shape

**Figure 6:**
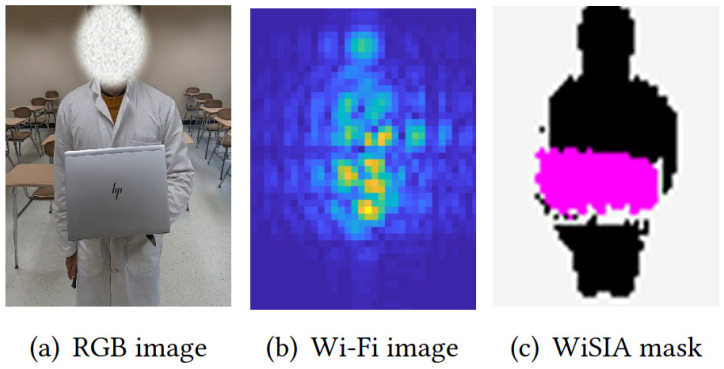
Example imaging results that were produced by WiSIA [50].

**Figure 7:**
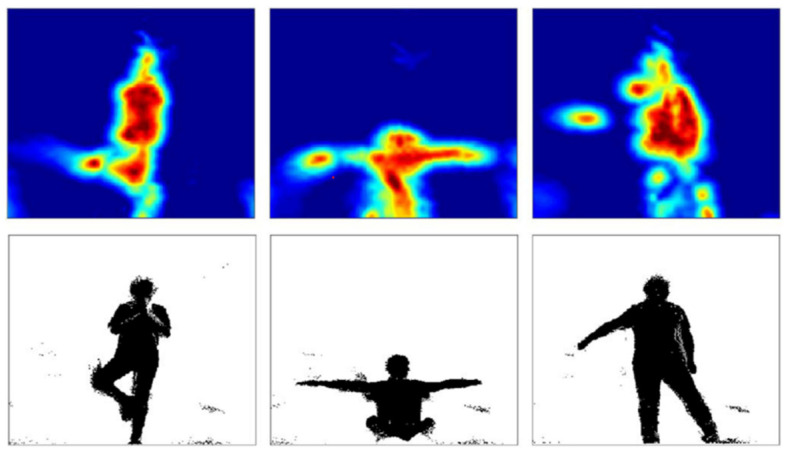
Example imaging results that were produced by 60 GHz Wi-Fi signals [76]. The upper and lower layers are images from the mmEye and Kinect cameras, respectively.

Currently, some excellent applications achieve contour imaging based on Wi-Fi CSI. For example, C. Li et al. [50] proposed WiSIA, a wireless imaging system enhanced by a generative adversarial network (GAN). WiSIA can simultaneously detect objects and humans, segment their boundaries, and identify them in an image, as shown in Figure 6. However, low-frequency signals have the disadvantage of narrow bandwidth. Wi-Fi millimeter-wave (mmWave) radar with a small number of antennas under 60 GHz has been used in commercial routers. It supports higher-resolution bandwidths, providing a new method for Wi-Fi imaging. The mmEye [76] is the first superresolution RF imaging system for a mmWave camera. It can utilize the sparsity of the target reflected signals in a single range, and design a new joint transmitter smoothing (JTS) based on the MUSIC algorithm. In addition, its imaging quality is close to that of the Kinect depth sensor, as shown in Figure 7.

#### 2.2.2. Wi-Fi CSI Skeleton

**Figure 8:**
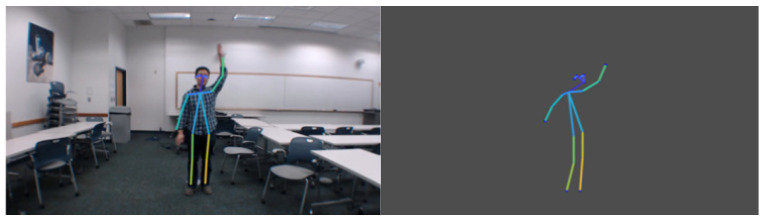
The Two-dimensional human skeleton is generated by 2.4 GHz Wi-Fi CSI [49]. The ground truth is shown on the left, and the CSI predicted skeleton is shown on the right.

**Figure 9:**
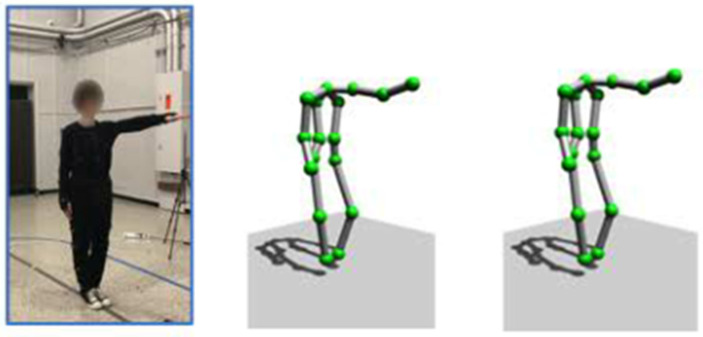
The three-dimensional human skeleton is generated by 5 GHz Wi-Fi CSI [51]. The images from left to right are the video frames, ground truth, and CSI predicted skeleton.

Recently, many studies have demonstrated that fine-grained wireless sensing can also be achieved using 1D sensors in the low-frequency band. Fei Wang et al. used 2.4 GHz Wi-Fi signals to generate 2D skeletons in [48,49], and achieved single-person and multiperson pose reconstruction. Figure 8 shows the single-person skeleton that was generated by [49]. Winect [77] combined signal separation and joint movement modeling to generate 3D human poses, enabling centimeter-level accuracy for free-form activity tracking. These articles [51,52] captured high-precision human body posture in 5 GHz Wi-Fi signals, shown in Figure 9. As shown in Figure 8 and Figure 9, the accuracies of the skeleton and the joint points that were generated by the former are not as high as those by the latter. According to the loss of wireless electromagnetic waves, the attenuation of 5 GHz will be faster with the increase in distance. Therefore, 2.4 GHz Wi-Fi signal has satisfactory wall penetration, and the 5 GHz Wi-Fi signal has poor wall penetration.

Based on the above research, we can conclude that the system’s performance based on the FMCW signal is almost the same as that of the camera. It can also protect the privacy and realize posture reconstruction when there is poor lighting. However, it requires dedicated hardware to collect signals and cannot adapt to most environments. The 60 GHz Wi-Fi radar overcomes the limitations of low-frequency RF imaging (the bandwidth, the number of antennas and the carrier frequency of 2.4 GHz/5 GHz Wi-Fi), but it is not as stable as the FMCW radar signal, which easily leads to signal response fluctuations. Low-frequency Wi-Fi signals (such as 2.4 GHz or 5 GHz) may not achieve the high precision of the FMCW and the 60 GHz Wi-Fi signals. They can be used in daily life to provide ordinary people with a full range of safety detection due to their advantages of having a low cost and an extensive deployment range.

## 3. Fundamental Knowledge of CSI Skeleton Recognition

Wi-Fi is a wireless local area network (WLAN) technology that is based on IEEE 802.11 standard protocols [78]. In contrast to Bluetooth and infrared sensors, Wi-Fi devices have a wide range of coverage. In the following section, we introduce the basic concepts of CSI and summarize the typical neural network models and the performance evaluation indices in CSI skeleton recognition.

### 3.1. Introduction to CSI

CSI, a typical radio frequency signal, can be calculated using Wi-Fi technology [24]. Orthogonal frequency-division multiplexing (OFDM) and multiple-input-multiple-output (MIMO) technologies in the 802.11 protocol are used to understand the transmission process of wireless signals. OFDM means that the signal is transmitted across multiple orthogonal subcarriers at various subcarrier frequencies. MIMO refers to the signal being transmitted with multiple antennas. The CSI can be extracted from the signals of a commodity Wi-Fi device that is equipped with a network interface card (NIC) [79]. It represents the channel properties of the communication link between the transmitter and the receiver. The signal that is received by the receiver can be expressed as follows:

(1) $$y_{i}=H_{i}x_{i}+\eta_{i}\;$$

where i is the subcarrier index; $x_{i}\in R^{N_{T}}\;$ is the transmitted signal; $y_{i}\in R^{N_{R}}$ is the received signal; $n_{T}$ and $n_{R}$ are the numbers of transmitter antennas and receiver antennas, respectively; $\eta_{i}$ is the noise vector; $H_{i}$ denotes the CSI matrix of subcarrier i as follows:

(2) $$H_{i}=\left(\begin{array}{cccc} {h_{i}}^{11} & {h_{i}}^{12} & \cdots & {h_{i}}^{1n_{T}}\\ {h_{i}}^{21} & {h_{i}}^{22} & \cdots & {h_{i}}^{2n_{T}}\\ \vdots & \vdots & \ddots & \vdots \\ {h_{i}}^{n_{R}1} & {h_{i}}^{n_{R}2} & \cdots & {h_{i}}^{n_{R}n_{T}} \end{array}\right)$$

where $h_{i}$ is the CSI of each subcarrier for the link between the receiver antenna and the sender antenna, and $h_{i}^{mn}$ is a complex value, which can be represented as:

(3) $$\;h_{i}^{mn}=\left|h_{i}^{mn}\right|e^{j∠h_{i}^{mn}}\;$$

where $\left|h_{i}{}^{mn}\right|$ and $∠h_{i}^{mn}$ denote the amplitude and phase, respectively. The CSI in (2) captures the status of the environment. NIC collects the CSI data on each subcarrier. Common CSI measurement tools are the Intel 5300 NIC [80], Atheros 9580 NICs [81], the Broadcom chipset [82], the ESP32 microcontroller [83] and the SDR [84].

### 3.2. Basic Description of the Skeleton

**Figure 10:**
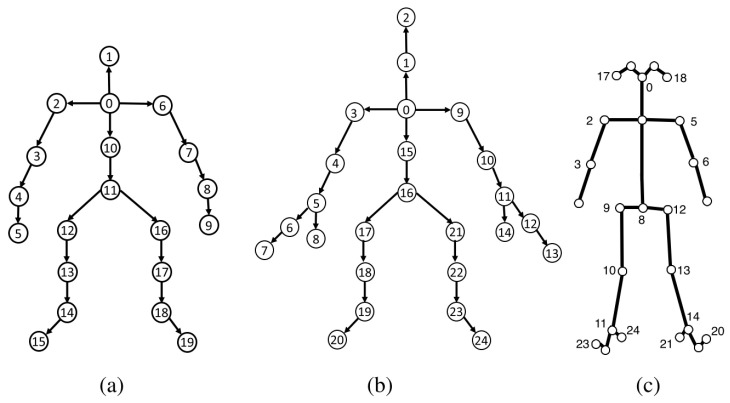
Human skeleton model by different techniques. (**a**): 20 joints (Kinect for Xbox 360), (**b**): 25 joints (Kinect V2) and (**c**): 25 joints OpenPose Body-25 methods [89].

- Skeleton-based action recognition has been widely used in computer vision due to its fast execution speed and strong ability to handle large datasets. Researchers can realize human action recognition by detecting changes in the position of joints, usually using techniques such as Kinect depth sensors (Xbox 360 Kinect, Kinect V2) [51,76,77,85], OpenPose [86], and Alphapose [87] to extract bone trajectories or poses.
- OpenPose and AlphaPose are the most commonly used algorithms in the process of generating human skeletons from images or video frames. OpenPose, which is a bottom-up approach, finds key points of the human body and joins them to the frame of the human [33,48,53,54,88]. In addition, it can estimate the human body and foot joints with the confidence score of each joint, e.g., 15 (OpenPose MPI), 18 (OpenPose-COCO), or 25 (OpenPose Body-25) keypoints, as shown in Figure 10c. In contrast, AlphaPose adopts the regional multiperson pose estimation (RMPE) framework to improve the pose estimation performance [49,52]. AlphaPose can estimate the 17 (Alphapose-COCO) or 26 (Halpe) key points of human joints. In CSI-based pose recognition, we usually choose a suitable method to extract the skeleton as the ground truth value.

### 3.3. Typical Neural Network Models

Currently, the deep learning model [90,91,92] is a widely recognized solution for extracting complex features from signals. It can automatically compose features at lower levels into features at higher levels to improve the accuracy of the prediction results. Since CSI does not contain direct information about human poses, human posture cannot be manually annotated in pose recognition based on Wi-Fi devices [33]. Therefore, we need to design a neural network model for extracting temporal and spatial information from Wi-Fi signals. Commonly used models are convolutional neural networks and recurrent neural networks.

Convolutional Neural Networks (CNNs): The convolution operation aims at reducing the dimensions of features in the calculation operation. It is usually used to extract the time and space features of Wi-Fi CSI and transform them into graphs. It is also broadly used in pose recognition [33,51,52,53,54,88]. Residual neural networks (ResNets) [93] are applied to image recognition tasks by introducing the concept of residual learning to CNNs. For example, ResNet [49,77] is used to extract useful data. CNNs play an important role in 3D pose recognition due to their satisfactory performances in high-dimensional data processing.

Recurrent Neural Networks (RNNs): RNNs use the time correlation among neurons to retain previous information in the current state. However, RNNs may cause the problem of gradient disappearance. Long short-term memory (LSTM) [55] solves this problem. It uses gradient descent as a memory unit to learn the characteristics of long sequences. Compared with direct convolution, the convolution of LSTM training is more effective and has the best performance in many-to-many imaging.

Hybrid models [55,94] contain features from more than two primary deep neural networks and they can help overcome the problems requiring many feature extractions. For example, the CNN + LSTM neural network is used for feature extraction [51] to extract deeper CSI information. With this approach, the extracted effective information is more comprehensive, and the generated skeleton is smoother. In the future, the design of new neural network models or the combination of neural networks for the extraction of complete and effective information will require further research. Furthermore, designing neural network models in different software development environments also has greater development potential. For example, Virtual Reality (VR) Platforms [95] can not only be used as learning tools, but also designed deep learning applications for human activity recognition.

### 3.4. Performance Evaluation Indicators

Pose construction requires the establishment of pose recognition models to generate human skeletons. In this section, we introduce the performance evaluation indicators of common pose recognition models, such as the percentage of correct keypoints (PCK), the mean per joint position error (MPJPE), the percentage of correct skeleton (PCS), and the average precision (AP). The mathematical formulas of PCK, PCS, AP, and MPJPE are presented as (4)–(7). Table 1 summarizes the applications of several evaluation metrics.

Percentage of Correct Keypoints (PCK): The PCK index measures the accuracy of body joint positioning. If the distance between the predicted joint and the real joint is within a specified threshold, the detected joint is considered to be correct. In PCK@a, a refers to either the length of the torso or the percentage of the length of the head. The higher the PCK value is, the better the performance of the system.

Average Precision (AP): AP is used to evaluate multiperson pose recognition. In the AP measure, the predicted joint is regarded as a true positive if it falls within the threshold of the ground-truth joint location. Moreover, for multiperson pose evaluation, all predicted poses are assigned to the ground-truth poses one-by-one based on the PCKh score order.

Percentage of Correct Skeletons (PCS): PCS refers to the percentage of the Euclidean distance that is below a specified threshold. θ < 25 indicates that human poses are accurate and complete, the position of the human body is correct in the predicted image, and the predicted image looks the same as the ground truth. θ < 30 means that the posture of the person is accurate. θ < 40 indicates that the position of the person is correct, but the posture is not accurate. θ < 50 means that as the number of limbs in the predicted image increases, the person’s posture becomes incomplete, blurred, and inaccurate. PCS ◦ 30 and PCS ◦ 50 indicate the strict matching and loose matching of the human body posture.

Mean Per Joint Position Error (P-MPJPE): P-MPJPE is the average value of the L2 distance between the predicted joint point and the corresponding ground-truth joint point. The smaller the index is, the higher the performance of the 3D human pose recognition method. However, these metrics treat each joint of the body independently and may not be able to assess the overall structure of the pose. In the future, further research is needed to propose new evaluation indicators or to combine them with previously established evaluation indicators in order to improve evaluation performance.

## 4. Pose Recognition Procedure

**Figure 11:**
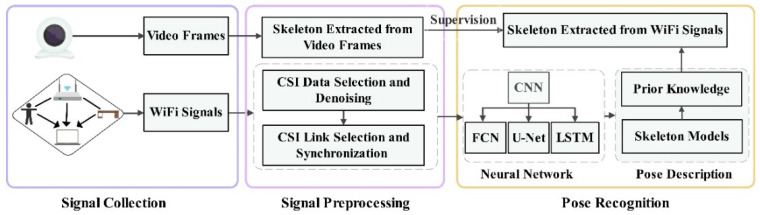
System overview of CSI pose construction.

To reconstruct a human skeleton using Wi-Fi devices, we need to collect CSI signals for data preprocessing, send the processed data into the model for training, and generate the human skeleton. This section introduces the process of pose estimation in detail using Wi-Fi CSI in four parts: signal collection, signal preprocessing, pose recognition methods, and system performance evaluation. The overall process of the system is illustrated in Figure 11. The complete system consists of the upper and lower parts. The upper pipe provides the ground truth for training supervision, and the lower pipe designs a neural network for extracting the time and space information of the CSI and predicting the posture of the human body.

### 4.1. Signal Collection

According to the Fresnel zone model [96], collecting pose information in the whole space need at least two pairs of transceivers accurately capture human pose graphics. Human pose information and human position information are contained mainly in the CSI amplitude and phase, respectively. To better understand the signal collection process, we research the signal collection process in the current HPR articles based on the Wi-Fi CSI. As presented in Table 2, we summarize them based on four aspects: experimental devices, signals and the preprocess method, the scene, and number of data.

### 4.2. Signal Preprocessing

In signal preprocessing, the raw CSI data measured by commodity Wi-Fi devices cannot be used directly for human pose recognition due to environmental noise, unpredictable interference, and outliers. The signal preprocessing method plays a key role in extracting human posture information. We summarize the general methods of signal preprocessing from two aspects: CSI link selection and denoising, and the synchronization of video frames with CSI samples.

#### 4.2.1. CSI Signal Characteristics Selection and Denoising

Amplitude: In CSI-based human pose recognition, it is essential to extract the information related to human pose from the amplitude of the CSI signal. The larger the action, the more obvious the amplitude change, and the higher the reconstruction accuracy of the human skeleton. The raw amplitude information extracted from CSI is not directly used. Currently, several amplitude denoising methods are available, such as principal component analysis (PCA), discrete wavelet transform (DWT), and Hampel filtering. PCA improves processing efficiency by reducing the dimensionality of the dataset, which can remove noise and data redundancy [33]. DWT can remove noise while extracting useful edge information [33,52,53]. Hampel filtering removes outliers caused by sudden changes in the device and the environment. It determines outliers through a moving average window and replaces them with the average of the data [33,55,88].

Phase: Phase is more sensitive to subtle changes in motion than amplitude characteristics. It is necessary to make full use of the amplitude and phase information of CSI for the robustness of the pose recognition system. However, consecutive CSI measurements can result in different time-varying phase offsets in data collection. Some articles [51,52] often adopt conjugate multiplication (CM) to eliminate the phase offset between two CSI antennas. In addition, Winect [77] used the blind source separation (BSS) [98] method to separate the phase changes of the signal and extracted the pose features.

Angle of arrival (AoA): The 2D AoA spectrum contains more spatial information about the human body. The 2D AoA of the reflected signal can be used to infer the number of limbs, enabling human pose reconstruction independent of the environment. However, in contrast to amplitude and phase, the 2D AoA derived from a single Wi-Fi device can only contain a small fraction of body motion. Therefore, multiple pairs of devices are required to combine the 2D AoA spectrum contained in multiple CSI packets [77,97]. In the process of inferring human pose from a 2D AoA spectrum, the phase variation of the CSI signal is typically separated first, and then the CSI amplitude is applied through a Fast Fourier Transform (FFT).

In conclusion, the amplitude, phase, and AoA spectrum of CSI contains different degrees of human motion information, and the accuracy of reconstructing human skeletons by selecting a certain feature or combining these three features may vary. Developing new key feature methods to obtain more pose information is an important direction for future development.

#### 4.2.2. Synchronization of Video Frames with CSI Samples

Since the pose features that are contained in CSI signals are relatively weak, first, we select the antenna with larger dynamic responses to obtain complete pose information. Papers [33,52,53,97] choose an antenna with the largest variance value as the reference.

Moreover, since the transceiver time is not synchronized, data may be lost, the transmission may be delayed, or the phase may be shifted; hence, the data collected by the camera cannot match the CSI samples. Generally, the camera’s number of frames per second (FPS) is much larger than the CSI sampling rate. The transceivers and the camera are synchronized using the network time protocol (NTP) to ensure that the CSI samples are synchronized with the video frames. Secure-Pose [88] uses linear interpolation to resample a group of fixed-interval CSI samples between two video frames to resolve these issues based on timestamps.

### 4.3. Pose Recognition Methods

The deep learning model can extract the spatial and temporal information of the CSI, locate key points of the body or the body parts, and further infer human poses in the image. This section summarizes the general framework of neural networks in CSI human pose recognition.

Networks Based on the CSI-Predicted Poses: In the process of generating the human skeleton with CSI, a neural network is designed mainly for feature extraction. We study all the articles on HPR based on Wi-Fi CSI and summarize them according to the classification of neural network models, as presented in Table 3.

A convolutional structure is adopted to encode the high-dimensional features into low-dimensional features [52,53,54,88], and extracts the features with the most useful information. In addition, resizing convolutions are used to eliminate the checker artifacts and decode the poses in the camera to realize pose reconstruction. CNN can substantially reduce model complexity while retaining robust feature extraction ability. ResNet [77] can train deeper convolutional neural networks to solve the degradation problem of traditional CNNs.

Hybrid models solve the limitations of a single neural network and can maximize the use of neural networks to solve real-life problems [48,49,51,55]. For example, Wi-Pose [51] includes four layers of CNNs and three layers of LSTMs on top of the CNNs. This model can encode prior knowledge of the human skeleton into the pose construction process, use LSTM to output the initial skeleton model, and obtain the current pose according to the forward kinematics.

In the field of neural networks, compared with 2D human pose recognition, 3D human pose recognition still needs to extract CSI spatial and temporal information. Hence, the convolution structure is crucial for the feature extraction process.

### 4.4. System Performance Evaluation

This section introduces the application of the loss function and the system recognition results based on pioneering papers in CSI human pose recognition.

#### 4.4.1. Loss Function Selection

Loss functions are used to optimize the model to minimize the difference between the predicted and ground-truth images. We summarize the commonly used loss functions and their applications in Table 4.

The binary cross-entropy loss function is used for the dichotomy task. Formula (8) solves for the probabilities of $D_{mk}$ and $\left(1-D_{mk}\right)$. Formula (9) is the average of the binary cross-entropy loss function of each pixel, where W refers to the number of pixels in the figure, and $P_{i,j}\;$ and $S_{i,j}$ relate to grayscale values that correspond to the $\left(i,j\right)$-th pixel in the image. Equation (10) represents the average Euclidean distance error, which is used mainly to predict the distance error between the predicted joint and the real joint. $\;{\tilde{p}}_{t}^{i}$ and $p_{t}^{i}$ are the predicted and true coordinates, respectively, of the joints at time *T*. The sum of the Huber loss and the average Euclidean distance error is used as a loss function optimization model [52]. ${\mathrm{pPAM}}^{x}$ and ${\mathrm{PAM}}^{x}$ are the prediction and supervision, respectively, of the pose adjacency matrix for human body key point coordinates on the *x*-axis. On the *y*-axis, ${\mathrm{pPAM}}^{y}$ and ${\mathrm{PAM}}^{y}$ have similar representations.

According to the formulas in Table 4, the most commonly used function is the L2 loss function. The calculation of this function is convenient, and the measurement error is low.

#### 4.4.2. System Recognition Results

This section discusses the system performances in HPR papers based on Wi-Fi CSI, as presented in Table 5.

Studies [48,49] could generate human poses, and the accuracy rates of the two systems exceeded 75% in identifying skeletons. Wi-Pose [51] could accurately locate the human position, and the accuracy rate was relatively improved. Compared with Wi-Pose, the study [33] had much higher accuracy and evaluated four different scenarios. DINN used [33] as the baseline comparison. In the visual and through-wall scenarios, the average percentages of DINN improved by approximately 37% and 35.7%, respectively, on PCS◦30.

Compared with Wi-Pose, the P-MPJPE value of Wi-Mose was smaller, indicating that Wi-Mose’s human skeleton precision was higher than that of Wi-Pose. However, Wi-Mose did not discuss cross-domain research. Wi-Pose used RFPose3D [68] as the baseline comparison. The average joint localization errors (unit: mm) of Wi-Pose were all smaller than those of RFPose3D in eight scenarios of various training rates, numbers of receiving antennas, occlusion, and cross-domain conditions. Wi-Pose was the most advanced deep learning model in 3D human pose construction for a series of predefined activities using Wi-Fi CSI.

Compared with Wi-Pose, Winect has higher tracking accuracy. Because it is designed to work with free-form activities, Wi-Pose is only used for a set of predefined activities. Free-form activities were more suitable for complex activities in daily life, but this system could only identify common activities at present, and further research is needed.

In this section, we summarize the process of human pose reconstruction by Wi-Fi CSI from four aspects: signal collection, signal preprocessing, pose recognition methods, and system performance evaluation. We analyze and compare algorithms in each aspect of these systems, which provides a complete pose research process for researchers. It will contribute to the future field of pose construction based on CSI.

## 5. Typical Models of Generating the Human Skeleton

In the following, we analyze the typical applications of three models based on skeleton models in pose recognition and discuss the advantages and disadvantages of each model.

### 5.1. Skeleton Models Based on the Human Silhouette

**Figure 12:**
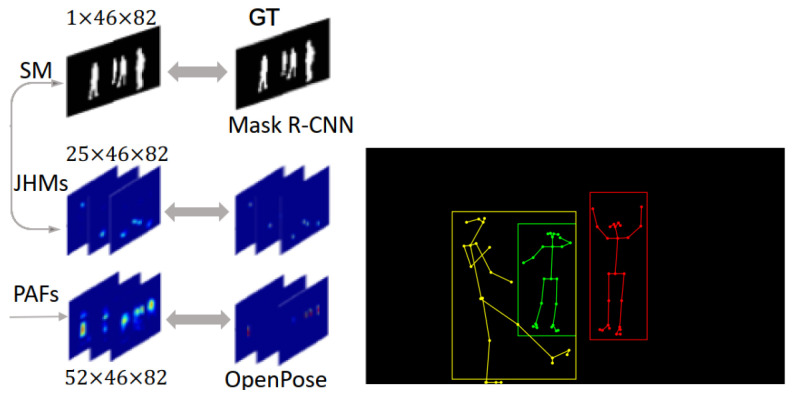
SM, JHMs, and PAFs are used for joint association to generate multiple person skeletons [48].

Silhouette models contain rough width and contour information of the limbs and torso. Human body parts are approximated by the rectangles or boundaries of human silhouettes and are usually computed by convolution. Generally, contours require preprocessing, i.e., segmentation masks (SM), to extract objects based on prior knowledge of the background. The extracted contour features are usually encoded as Fourier descriptors, geometric features, shape contexts, etc. Furthermore, silhouette-based models commonly use heatmap methods to generate human skeletons [48,55,88]. For example, [48] used Mask R-CNN to map CSI tensors to SM, Joint Heat Maps (JHMs), and Partial Affinity Fields (PAFs). JHMs refer to confidence maps of key positions of the body, and PAFs encode the positions and the orientations of the limbs. Figure 12 shows a neural network model that transforms CSI variables into SM, JHM, and PAF variables to generate human contours and the final human skeleton, which is more suitable for multi-person pose recognition. The heatmap methods directly return each type of key point’s probability, providing supervision information for each point.

### 5.2. Skeleton Models Based on Key Point Coordinate Regression

In contrast to the heatmap method, regression key point coordinates need to extract all possible body key points from CSI pose images, encode the positions and directions of human limbs, and connect parts in the same body. The model uses the coordinates (x, y) of the target point as output to generate the human skeleton. Scholars have published three papers based on this model, namely, Wi-Pose [33,53], and Wi-Mose [52]. However, this model, based on key point coordinate regression, lacks the width and contour information of the human body. As a result, skeleton migration may not accurately generate skeleton images when facing new environments and new people.

**Figure 13:**
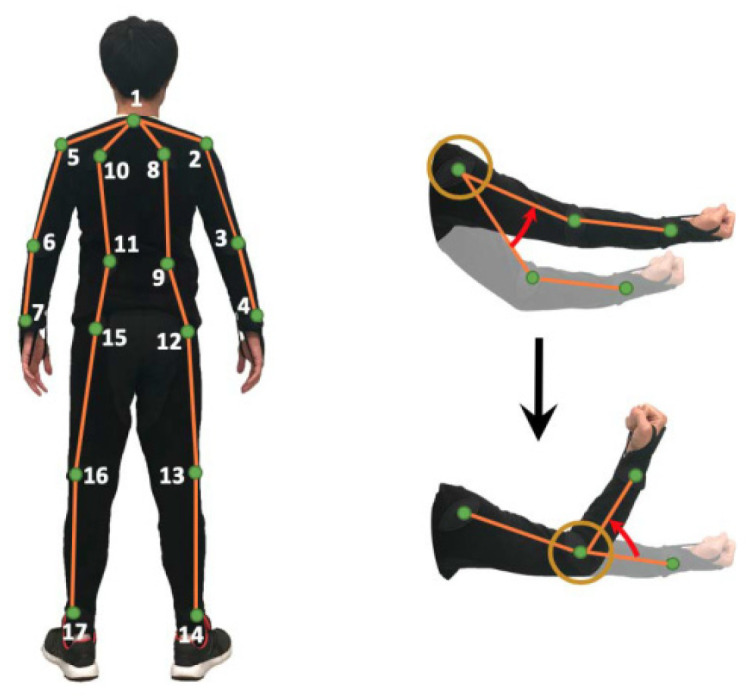
The left picture shows the skeleton tree model proposed in [51], and the right picture shows the joint rotation. The elbow is relative to the shoulder and rotates the hand with respect to the elbow.

To overcome the disadvantages of regression key points, some authors proposed another model [51] based on skeletons. The model treats a skeleton as a tree, with the nodes being the joints and the edges being the body segments. In the skeleton tree, because the lengths of the limb parts are fixed, the position of each joint can be inferred simply by estimating the rotation of its associated body segment with respect to the parent joint. Figure 13 shows the movement of an arm. The shoulder is the parent joint of the elbow, which is also the parent joint of the hand.

The human skeleton model based on the tree structure of joints makes up for the shortcomings of the direct regression key point model. This model is designed to learn the rotation of the joints and use the time information to infer the current motion pose to ensure that the joints can naturally satisfy the constraints of the bones. This model can not only be applied in human pose recognition, but also in robotics and/or exo-suit engineering applications, to detect the user’s movement and direction, and recover and track the arm. In robotics and/or exo-suit engineering applications, IMU (Inertial Measurement Unit) devices, such as gyroscopes and accelerometers, should be employed.

### 5.3. Skeleton Models Based on the Point Cloud

To overcome the limitation of the key point coordinate model, researchers have proposed another solution: using various data analysis algorithms to extract as much human spatial information as possible from CSI signals to reconstruct human posture. Specifically, Winect [77] combined AoA and point cloud methods, extracted limb and joint information from CSI signals, and reconstructed 3D human posture.

Angle-of-arrival (AoA) is a typical ranging-based location algorithm; that is, a linear antenna array is used to infer the angle of arrival (i.e., the elevation angle and the azimuth angle) of the received signal in the N-dimensional range. The point cloud is mainly the scattered digital data measured by 3D scanning equipment, which can construct the model of the measured object. Winect estimates the 2D angle of arrival from the measured CSI data, and then analyzes the signal power change in 3D space based on the derived 2D AoA spectrum. In addition, the system uses the density-based spatial clustering of applications with noise (DBSCAN) algorithm to identify the number of moving limbs and the specific limbs in motion according to peaks in the azimuth-elevation spectrum.

**Figure 14:**
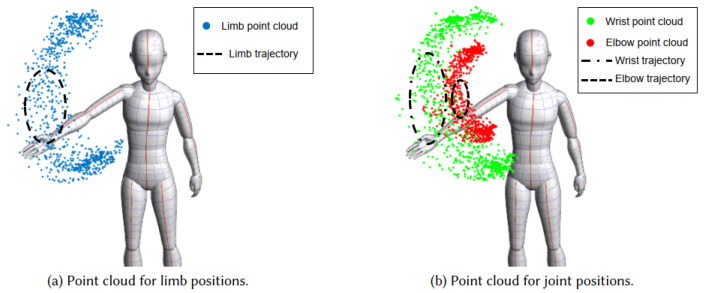
Examples of point clouds reconstructing limb and joint positions.

**Figure 15:**
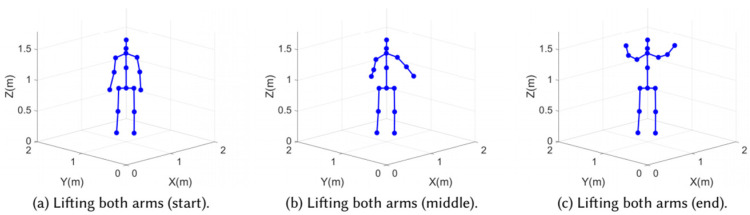
(**a**–**c**) show the continuous 3D skeleton: the process of lifting both arms.

Winect uses the number of identified moving limbs, and then separates the multi-limb motion signals using the BSS method. Then, Winect calculates the limb position based on the path length change of the separated signals and reconstructs the trajectory of the limb. Then, the system solves the joint positions using the kinematic model of the limb joints and builds the point cloud model to solve for limb and joint positions, as shown in Figure 14. Both limb and joint clouds are input to the ResNet network to predict the positions of multiple joints. Winect extracts joint and limb information from CSI signals and reconstructs limb trajectories using AoA and point cloud algorithms. Figure 15 shows the 3D human skeleton generated by Winect’s point cloud model. Deeply mining the available information of CSI, signals provides a variety of solutions for human pose reconstruction with commodity Wi-Fi devices.

This section introduces three models for generating the human skeleton, which are silhouette-based, key point coordinate-based, and point cloud-based human models. Table 6 describes the applications of the models for generating the human skeleton, the process of converting CSI data into a skeleton, and the advantages and disadvantages of each model. In general, the heatmap model may lose accuracy due to the slow speed of training and reasoning. The model based on the direct regression of key points lacks spatial generalization. Combining the description of prior knowledge and the tree structure based on the joint model renders the reconstructed human posture more realistic.

Facing different experimental scenarios, we may choose the appropriate skeleton model that can greatly improve the system’s performance. When the CSI data change is not obvious due to many interference items in the experimental scenarios, the key point coordinate regression model may not obtain good accuracy. At this time, the silhouette-based model can first locate the human body position and eliminate the interference items. In this case, the silhouette-based model, although slower to train, will perform better. The skeleton model based on point cloud is more suitable for recovering the high-speed movements of human body parts, but it cannot separate the reflection of complex signals. In the following pose recognition research, combining the skeleton model with data analysis algorithms can extract more human body information and adapt to more complex environments.

**Table 6:** Typical Models of Generating Human Skeleton.

Models	Applications	Generating Skeleton Process	Strength	Weaknesses
Human Silhouette	[48,55,88]	CSI Data -> Segmentation Masks -> HeatMap (JHM and PAF) -> Silhouette -> Skeleton	This model has satisfactory spatial generalize ability and improves the positioning accuracy of key points.	Slow training and inference.
Key Point Coordinate	[33,49,52,53,54]	CSI Data -> Key Point Coordinate -> Skeleton	This model has the advantage of fast training speed.	Poor spatial generalization.
	Skeleton Tree: [51].			
Point Cloud	[77,97]	CSI Data -> Phase: AoA Estimate -> Joint Trajectory: Point Cloud -> Skeleton	This model combines a variety of data analysis algorithms to fully separate the CSI signals.	Unable to solve complex signal reflection separation in dynamic scenes.

## 6. Discussion

This section discusses the influence of the experimental factors on the system’s robustness. It presents some insights into the development of human pose recognition from two categories, namely, experiential scenario and recognition user. In particular, we intend to investigate these typical applications and emphasize their crucial characteristics. We hope this discussion can provide useful insight for developers.

### 6.1. Experimental Scenarios

The current model works well indoors since the related experiments are usually conducted under a single indoor scene. Due to the influence of the experimental background, lighting, and other factors, the accuracy of CSI skeleton prediction will decrease when the environment changes. In addition, the CSI signal of Wi-Fi devices has the multipath effect and the packet rate also has an impact on the system identification accuracy. Therefore, we discuss the effects of the following on human pose recognition: whether the experimental scene is line-of-sight (LoS) or non-line-of-sight (NLoS), and whether the cross-domain scene can generate skeletons and the deployment of Wi-Fi devices.

#### 6.1.1. LoS or NLoS

Pose recognition that is based on CV cannot reconstruct poses in NLoS scenes, but Wi-Fi signals can penetrate occlusions to recognize skeletons. We compare the pose construction in the LoS and NLoS scenarios, as presented in Table 7.

Here, the Occluded Scenario and Through-wall Scenario refer to NLoS scenes. Obstacles (wooden screens or iron doors) are used to block the propagation and reflection of wireless signals. When signals pass through obstacles, the pose recognition error increases due to signal attenuation. Compared with the LoS scenes, the overall performances for the NLoS scenes are slightly reduced. CSI is still unable to generate a human skeleton through a wall. The realization of high-precision pose recognition after the signal passes through the wall is a major direction for future research.

#### 6.1.2. Robustness Discussion: Cross-Domain Scenarios and Stranger Situations

Recently, many studies have demonstrated that wireless network devices can be used to reconstruct images of human poses, producing satisfactory results in constructing images of subjects in prior training samples. However, in the transmission of the process, Wi-Fi signals may be reflected and scattered by objects in the surrounding environment. Moreover, subjects of various ages, genders, heights, and weights can affect the signal differently, even if they take the same action. Therefore, the performance will decrease for new subjects or testing subjects who are not in the training sample.

Currently, several solutions are available for improving cross-domain generalization ability. One solution is to add an adversarial neural network to extract domain-independent features [54]. The other is to improve the generalization ability at the lower signal layer [51].

The more robust the system is, the greater the possibility of large-scale applications. Hence, in the future, we should design new models to address these problems and accurately extract features independent of the environment to realize cross-environment generalization.

#### 6.1.3. Different Distances and Packet Rate of Wi-Fi Devices

The distance between the Wi-Fi transceivers has essential effects on skeleton recognition accuracy. Due to the multipath of the CSI signals, the system performance will be significantly improved with the decrease in distance. Besides the distance between Wi-Fi devices, the rate at which the devices themselves send packets also has an impact on the system’s performance. The sample size of the received CSI will change with the packet rate, as shown in Table 8.

Table 8 summarizes the average localization error (cm) changes of the human key points at different distances and packet rates in two typical papers [77,97]. As the distance between the two transceivers increases, the performance of the system significantly decreases. However, if the distance is too small, it is difficult to adapt to the long-distance application. Generally, the default packet rate is set as 1000 pkts/s, because high packet rate can effectively recover the joint movement of a body part and can better represent the full-body movements of a user. According to the current experimental results, the minimum packet rate is 250 pkt/s. Therefore, the appropriate distance and packet rate should be selected according to the actual target needs.

**Table 8:** Effects of Different Distance and Packet Rate on System Performance.

System	Distance; Metric Performance	Packet Rate; Metric Performance
Winect [77]	2 m; 4.6 cm	250 pkts/s; 5.7 cm
	2.5 m; 4.9 cm	500 pkts/s; 5.0 cm
	3 m; 5.1 cm	1000 pkts/s; 4.5 cm
	3.5 m; 5.4 cm	-
GoPose [97]	2.5 m; 4.7 cm	250 pkts/s; 5.7 cm
	3 m; 5.1 cm	500 pkts/s; 5.3 cm
	3.5 m; 5.8 cm	1000 pkts/s; 4.7 cm

In Table 8, Metric Performance is average localization errors (cm).

### 6.2. Recognition User

The object of posture reconstruction is a human. Therefore, the user’s status has an important impact on pose recognition, which includes three main aspects: 2D–3D, signal-multiple person, and static-dynamic posture. We analyze and summarize the advantages and the disadvantages of each category, providing a preliminary research direction for research.

#### 6.2.1. 2D or 3D HPR

2D HPR refers to the extraction of a 2D human skeleton by locating the coordinates of the human body’s key points (x, y) and connecting these key points in a specified joint order [33,48,53,54]. 3D HPR refers to the extraction of a 3D human skeleton obtained by locating the joint’s 3D coordinates (x, y, z) and connecting these key points in a specified joint order [51,52].

Two main methods are available for 3D HPR: one method is to obtain the 3D key points directly from 2D images through regression and design a neural network to realize end-to-end mapping. The other method is to predict the 2D pose from an image and predict the 3D pose and the trajectory of the root node based on the predicted 2D skeleton. The latter approach benefits from mature 2D pose recognition technology, which greatly reduces the complexity. However, substantial challenges are encountered with 3D human pose recognition, such as the huge 3D pose space and the inadaptability of single-view 2D to 3D mapping (for example, one 2D skeleton can correspond to multiple 3D skeletons). However, it is more important for developing intelligent medical care and motion analysis, among other applications. These problems must be urgently solved if we seek to obtain the real human posture.

#### 6.2.2. Single-Person or Multiperson HPR

Human pose recognition can be divided into two categories: single-person pose estimation (SPPE) and multiperson pose estimation (MPPE). SPPE involves the location of the key points of a person through CSI pose images to generate a human skeleton.

MPPE requires the determination of the key points of all people in CSI pose images of multiple people [48]. Since the positions and the number of people in the figure are unknown, MPPE is more difficult. We can draw experience from CV processing methods, such as the top-down or bottom-up method. The top-down approach requires adding a human detector, estimating each body component separately, and finally calculating the posture of each person. The bottom-up approach involves detecting all the components in the image and associating each component with a person. In general, adding body detectors is easier than using correlation algorithms.

#### 6.2.3. Static or Dynamic HPR

Static-based human behavior recognition refers to obtaining the corresponding effective information from a CSI posture image to realize the recognition of activities. Static behavior recognition involves only estimating the position of the skeleton and the coordinate position information of the N joint points of the human body that correspond to the skeleton at each moment [33,48,53,54].

Dynamic-based human behavior recognition requires the establishment of a relationship between the previous frame and the next frame in a video and the recognition of activities [51,52]. Movement refers to continuous actions that can form daily activities, such as walking and waving. In dynamic pose recognition, pose continuity must be considered because joint point changes adhere to the human body structure. An LSTM network is commonly used because it considers the effect of time on the joint points. In recent years, static posture estimation based on Wi-Fi devices has yielded better results. In contrast, dynamic posture estimation is more challenging.

In summary, we discuss and analyze the importance of current system recognition accuracy and system robustness for large-scale pose recognition in different scenarios. Next, we present some challenges and propose future research trends.

## 7. Future Research Trends

This survey provides a systematic overview of human pose construction and behavior analysis based on Wi-Fi CSI and conduct a comprehensive classification and performance comparison of human pose recognition applications. Although great progress has been made, many challenges remain. The section classifies these challenges from two aspects: key techniques and potential application scenarios. Moreover, we highlight promising directions to promote the development of HPR research.

### 7.1. Technique Transfer from Computer Vision

Human pose recognition based on computer vision has developed rapidly, and pose recognition techniques have achieved amazing progress. Transferring vision-based pose recognition algorithms to pose recognition based on CSI is a potential solution due to the similarity between recognition applications. This section introduces the transfer of vision-based algorithms to CSI-based algorithms from two aspects: neural network structure and pose description.

#### 7.1.1. New Neural Network Structure

Human behavior can cause complex effects on CSI signals. Therefore, feature extraction from CSI using a neural network is the fundamental task of the system to effectively realize accurate behavior recognition. However, the corresponding neural network models are relatively few due to the lack of wireless pose recognition research. CSI-based pose recognition and vision-based pose recognition have various similarities; hence, wireless pose perception can adopt neural network models from CV pose recognition (e.g., DensePose-RCNN [99], OpenPose, AlphaPose). Paper [49] uses the multistage CNN model to predict body parts’ positions to generate a 2D confidence map, which proves that the neural network model of a transfer learning CV is feasible. We can utilize the successful neural network model in CV (i.e., Graph Neural Network [100,101] and Transformer [102,103], or mixed [104], Attention [105]) to implement skeleton recognition using CSI.

In addition, we believe that the following learning strategies for CSI pose recognition will work well because they have been used successfully in the classical CV models. Human pose recognition can be transformed into target recognition and human posture, and key point coordination can be obtained from the image using CNN [106]. The coarse-to-fine method [107] can also be learned to resolve the problems of the block, invisible key points, and complex background. Specifically, crucial key points (e.g., head, hands, feet) can be detected initially, and then the other key points can be calculated by using all feature representations and data mining (elbow joints and knee joints). Technique transfer from the CV-based pose recognition model may play an important role in the future development of CSI pose recognition. We can also acquire many successful experiences from CV and design an effective neural network model to implement skeleton recognition using CSI.

#### 7.1.2. Realistic Pose Model and Complex Activity Description

**Figure 16:**
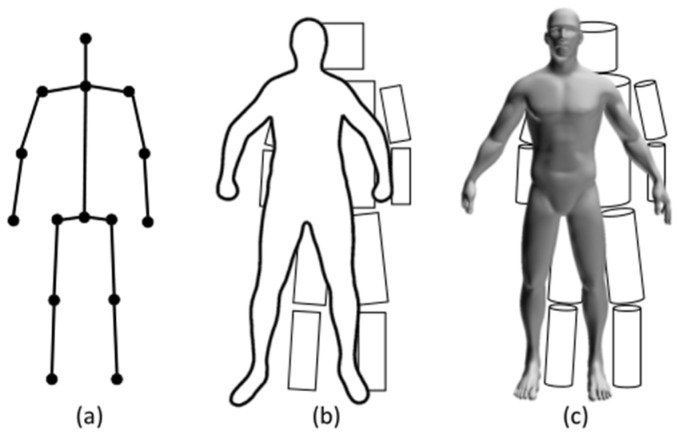
Three commonly used types of human body models in CV [113]: (**a**) skeleton-based models; (**b**) contour-based models; and (**c**) volume-based models.

At present, the basic research areas of human action recognition include human motion recognition and action recognition, which are less difficult than activity analysis [89]. Therefore, defining a suitable posture description and an accurate relationship between the spatial and temporal features is the primary problem we face [108]. In addition to the skeleton-based model, there are two types of commonly used human body models in CV: contour-based models and volume-based models, as shown in Figure 16. When we identify human action, we should consider suitable pose descriptions, including the effect of body deformation [109]. The skeleton-based model is widely used in CSI pose description, but it may still lack sufficient information to depict complex human poses to implement the accurate and real human activity. Therefore, we can utilize the person’s prior knowledge, such as the structural feature of the pose [110], the kinematic dependency between the keypoint and skeleton [111], and the motion feature of a human body [112], to render the generated human poses as realistic. We can choose a suitable model for pose recognition according to the requirements.

Due to the complexity of activities, they may be divided into a series of pose sequences. At the same time, posture can be described as a skeleton. As a result, the representation of skeleton sequences for action recognition becomes a very important problem [114]. Therefore, pose recognition for action recognition has become a trending research topic [115]. When considering human action, we should choose the representation with more spatiotemporal features [116] to depict skeleton sequences accurately. In addition, different views of the action may be very different [117]. Identifying several successive postures is always challenging when we want to understand subtly different actions [118]. Understanding both high-level semantics and internal temporal structures of actions is a more complicated problem in CV research [119]. In summary, defining skeleton sequences to describe human action and understand subtle action differences is crucial for human behavior recognition using CSI. We can obtain successful experiences from CV studies.

### 7.2. Future Application Scenarios

The current studies achieve some research results about multiple people, 3D postures, and dynamic actions using video techniques [120]. The typical applications include action recognition, online coaching, entertainment, health care, etc. This section considers the following two typical human skeleton application scenarios based on CSI, health care, and public area monitoring. They are potential applications because these two scenarios may involve many rooms, requesting a through-the-wall environment, or concern privacy violations at public area monitoring. Traditional skeleton recognition applications using video techniques may not work well under these scenarios.

#### 7.2.1. Comprehensive Elderly Family Health Monitoring

Long-term and accurate human action monitoring is very important for patients or single old adults because it can provide health conditions in time. Currently, some studies have proposed health monitoring using CSI signals at home or hospital due to its important advantages [121]. For instance, WiMonitor [122] proposes monitoring the health of elderly individuals who live alone, which can realize status tracking and instantaneous abnormal responses for several months. At the same time, some video applications using the human skeleton are also employed in health care scenarios and achieve important research results [123,124,125]. Therefore, health monitoring using human skeletons based on CSI is a potential research topic. For example, Guo et al. [126] used a skeleton to monitor elderly individuals for abnormal behavior (i.e., falls) based on CSI information. In addition, combining multiple functions (such as family location, posture recognition, and respiratory monitoring) to realize long-term family health monitoring and abnormal health status alarms is a large requirement for medical health.

Human skeleton tracking technology based on Wi-Fi signals has the advantages of easy deployment and through-the-wall monitoring in the indoor environment, which effectively solves the problems of traditional health monitoring systems. In addition, how to deal with pose recognition under changing environments can also help elderly individuals living alone or dementia patients obtain health assistance in time. These applications prove the necessity and urgency of skeleton tracking technology using CSI.

#### 7.2.2. Behavior Recognition in Public Areas

Currently, cameras are installed in many scenarios to record public information [127]. However, these devices may cause violations of personal privacy if they are misused. Some governing bodies are implementing stricter usage and management requirements for these devices. In contrast, surveillance using CSI can effectively provide activity recognition and behavior analysis without privacy violations [128]. At the same time, some video applications utilize the human skeleton to implement posture recognition and activity analysis without privacy issues [129]. Therefore, we can utilize skeleton recognition using CSI techniques to realize precise pose recognition in a public area. As a result, precise human pose recognition based on CSI and the human skeleton may be a satisfactory solution to these problems. For example, we can analyze customer behavior using CSI without video records. This method can obtain similar performance without any privacy violations.

Furthermore, the combination of penetrating wall features of CSI and precise human skeleton recognition will open the door to these potential applications. Moreover, many applications that are related to video surveillance projects can be replaced due to the excellent work characteristics of CSI and skeleton models.

## 8. Conclusions

In this work, a survey of recent studies on human pose recognition using pervasive Wi-Fi CSI signals is conducted. This paper presents a general framework of precise behavior recognition based on the human skeleton and CSI, as well as summarizes the main components, including the signal processing techniques, neural network models, and performance results of pose recognition. In addition, we analyze the typical applications of pose recognition according to various skeleton models and discuss typical factors from the experimental environment to potential applications. We believe that these analyses and discussions help widen the vision of researchers and enable them to learn more mature experiences from these applications. Furthermore, we deem that transferring computer vision algorithms to CSI pose recognition may be a promising strategy for solving many difficult problems. We also believe that the combination of precise skeleton recognition, localization, and complicated human behavior analysis using CSI will enable us to develop novel applications.

## Figures and Tables

**Table 1:** Common Evaluation Metrics.

Evaluation Metric	Application	Formula	
Percentage of Correct Keypoints (PCK) PCK@a	[48,49,88]	$PCK_{i}@a=\frac{1}{N}\overset{N}{\underset{i=1}{\sum }}I\left(\frac{∥pd_{i}-gt_{i}{∥}_{2}^{2}}{\sqrt[{2}]{rh^{2}+lh^{2}}}⩽a\right)$	(4)
Average Precision (AP)	[48]	$\mathrm{AP}@a=\frac{1}{N}\overset{N}{\underset{n=1}{\sum }}I\left(100\cdot IOU_{n}\geq a\right)$	(5)
Percentage of Correct Skeletons (PCS) PCS ◦ θ	[33,53,54,55]	$PCS^{\circ }\theta =\frac{1}{N}\overset{N}{\underset{n=1}{\sum }}L\left(∥p_{i,j}^{n}-g_{i,j}^{n}∥{}_{2}⩽\theta \right)$	(6)
Mean Per Joint Position Error (P-MPJPE)(mm)	[51,52]	$E_{MPJPE}\left(f,S\right)=\frac{1}{N_{S}}\overset{N_{S}}{\underset{i=1}{\sum }}∥m_{f,S}^{\left(f\right)}\left(i\right)-m_{gt,S}^{\left(f\right)}\left(i\right){∥}_{2}$	(7)

**Table 2:** Device, Scene, and Data for Signal Collection.

System	Device	Signals and Preprocess	Experiment	Number of Data
WiSPPN [49]	5300NIC; one sender and one receiver; 2.4 GHz frequency with 20 MHz.	Amplitude	Eight volunteers, daily actions in two rooms of the campus, one laboratory room and one classroom.	80 k images, training and testing sample sizes are 79,496 and 19,931; 80%: training; 20%: test.
Person-in-Wi-Fi [48]	5300NIC; three receiver antennas, 2.4 GHz, 20 MHz.	Amplitude	Eight volunteers, one laboratory room and one classroom; 100 Hz from receiver antennas and videos at 20 FPS.	Training and testing sample sizes are 123,631 and 30,996; 80%: training; 20%: test.
Wi-Pose [53]	5300NIC; 3 commodity Wi-Fi devices (one transmitter and two transceivers); 5 GHz, 20 MHz.	Amplitude; DWT	Five volunteers, environment is approximately 7 m × 8 m; CSI at 100 Hz and videos at 20 Hz;	1,800,000 CSI samples at each receiver; 75%: training; 25%: test.
[33]	5300NIC; 3 commodity Wi-Fi devices; 5 GHz, 20 MHz.	Amplitude and phase; PCA; DWT; Hampel Filtering.	CSI at 100 Hz, and the video collection frequency at 20 Hz.	-
DINN [54]	5300NIC; 5 GHz frequency band with 20 MHz.	Amplitude	7 m × 8 m	10 h of data for 5 people; 5,400,000 CSI samples at each receiver; 75%: training; 25%: test.
Wi-Pose [51]	One laptop and three desktops, 5300NIC; 21 VICON Vantage cameras; 5.825 GHz; 20 MHz.	Amplitude and phase; conjugate multiplication (CM).	Ten volunteers; CSI at 1000 Hz, and the video collection frequency at 10 Hz.	70%: training; 30%: test.
Wi-Mose [52]	Three commodity Wi-Fi devices; 5 GHz; 20 MHz.	Amplitude and phase; CM.	Five volunteers; the environment is approximately 7 m × 8 m; CSI data at 150 Hz and video frames at 30 Hz.	10 h of data for 5 people; 5,400,000 CSI samples at each receiver; 75%: training; 25%: test.
Secure-Pose [88]	A Logitech 720p camera and Intel 5300 NICs; 5.6 GHz; 20 MHz.	Amplitude; Hampel Filtering.	Five volunteers; environment is approximately 8 m × 16 m; CSI at 100 Hz, and the video collection frequency at 20 Hz.	12,000 CSI samples
Winect [77]	Five laptops: one transmitter, four receivers; 5.32 GHz; 40 MHz.	Amplitude and phase; BSS.	Six volunteers (3 males and 3 females); one living room and bedroom.	Each volunteer performs various activities for at least 20 min.
[55]	5300NIC; 3 commodity Wi-Fi devices; 5 GHz, 20 MHz.	Amplitude; Hampel Filtering.	Five volunteers, environment is approximately 7 m × 8 m; CSI at 100 Hz and videos at 20 Hz.	75%: training; 25%: test.
mmEye [76]	Qualcomm 802.11ad Chipsets; 60 GHz frequency band with 3.52 GHz.	Amplitude and phase	Four volunteers; a typical office is approximately 28 m × 36 m.	Each subject performs 10 to 15 different postures for approximately 30 s.
GoPose [97]	Five laptops: one transmitter, four receivers; 5.32 GHz; 40 MHz.	AoA	Ten volunteers; a living room (4 m × 4 m), a dining room (3.6 m × 3.6 m), and a bedroom (4 m × 3.8 m).	The total time span of data collection is one month.

In Table 2, 80%: training refers to 80% of the data being used to train the network, and 20%: test refers to 20% of the data being used to test the network.

**Table 3:** Neural Network and Training Details.

Model	System	Neural Network Implementation	Training Details	Output
CNN	Wi-Pose [53]	Encoder network: three layers (3 × 3 convolutions), one layer (1 × 1 convolutions) and a fully connected layer. Decoder network: two layers (1 × 1 convolutions) and five layers (3 × 3 convolutions).	Adam optimizer; learning rate: 0.001; batch size: 16.	Key point coordinates
	[33]	Encoder network: three layers (3 × 3 convolutions), three layers (1 × 1 convolutions), a fully connected layer and an SE block. Decoder network: the same structure as in [53].	Adam optimizer; learning rate: 0.001; batch size: 16.	Key point coordinates
	Wi-Mose [52]	Feature network: 13 residual blocks and a batch normalization layer. Key-point regression network: two fully connected layers	Adam optimizer. The initial learning rate is 0.001, which is multiplied by 0.9 after every epoch.	Key point coordinates
	DINN [54]	Feature extractor, generator and domain discriminator.	Two Adam optimizers. One optimizes the feature extractor and generator networks: 0.001, the other optimizes the discriminator network: 0.0001.	Key point coordinates and the predicted domain
	Secure-Pose [88]	CSI transformer, JHM generator and PAF generator.	The batch size is 1, and an RMSprop optimizer with a weight decay of 1 × 10^−8^ and momentum of 0.9 is used. The CSI2Pose network: 1 × 10^−6^, the detection network: 1 × 10^−5^.	JHMs, PAFs
	Winect [77]	ResNet 18: 17 convolutional layers and one fully connected layer.	Adam optimizer, dropout rate is 0.1.	Key point coordinates
Hybrid model	Person-in-Wi-Fi [48]	CNN + U-Net	Adam optimizer; learning rate: 0.001; batch size: 32.	JHMs, PAFs
	WiSPPN [49]	ResNet + CSI-Net + FCN	Adam optimizer; Initial learning rate of 0.001, which decays by 0.5 at the 5th, 10th and 15th epochs; a batch size of 32.	Key point coordinates
	Wi-Pose [51]	CNN + LSTM	-	Key point coordinates
	[55]	LSTM + 3D-CNN	Adam optimizer; Initial learning rate of 0.0002; an ϵ numerical stability parameter is 1 × 10^−8^.	JHMs, PAFs
	GoPose [97]	CNN + LSTM	-	Key point coordinates

**Table 4:** Loss Functions Selection.

Loss Functions	Applications	Formula	
Binary Cross Entropy Loss	[48,54]	$\begin{array}{c} L_{d}\left(d,D\right)=\frac{1}{M}\overset{M}{\underset{m=0}{\sum }}\overset{K}{\underset{k=0}{\sum }}D_{mk}log\frac{1}{d_{mk}}\\ \;\;\;+\left(1-D_{mk}\right)log\frac{1}{1-d_{mk}} \end{array}$	(8)
Average of Binary Cross Entropy Loss	[39,53]	$ℒ=-\frac{1}{W}\underset{i,j}{\sum }S_{i,j}\log P_{i,j}\;+\left(1-S_{i,j}\right)\log\left(1-P_{i,j}\right)$	(9)
Average Euclidean Distance Error	[51,52,77]	$L_{P}=\frac{1}{T}\overset{T}{\underset{t=1}{\sum }}\frac{1}{N}\overset{N}{\underset{i=1}{\sum }}∥{\tilde{p}}_{t}^{i}-p_{t}^{i}∥{}_{2}$	(10)
Mean Squared Error (MSE) Loss	[55,88]	$\underset{{ℱ}_{w}}{min}\overset{\gamma }{\underset{y=1}{\sum }}{ℒ}_{JHM}\left(S_{1}^{y},S_{w}^{y}\right)+{ℒ}_{PAF}\left(L_{I}^{y},L_{W}^{y}\right)$	(11)
Pose Adjacent Matrix Similarity Loss	[49]	$ℒ=∥{\mathrm{pPAM}}^{x}-{\mathrm{PAM}}^{x}∥{}_{2}^{2}\;\;\;\;\;+∥{\mathrm{pPAM}}^{y}-{\mathrm{PAM}}^{y}∥{}_{2}^{2}$	(12)
Dice Loss	[88]	$D\left(P,Q\right)=1-\frac{2\sum_{x,y,z}\left|p\left(x,y,z\right)\right|\cdot \left|q\left(x,y,z\right)\right|}{\sum_{x,y,z}\left|p\left(x,y,z\right)\right|+\left|q\left(x,y,z\right)\right|}$	(13)

**Table 5:** The System Performance of HPR Based on Wi-Fi CSI.

Model	Metric Performance
WiSPPN [49]	PCK@a; PCK@5,0.04;PCK@10,0.14;PCK@20,0.38;PCK@30,0.59;PCK@40,0.73;PCK@50,0.82.
Person-in Wi-Fi [48]	PCK@0.2 PCK@0.2,78.75%
Wi-Pose [53]	PCS ◦ θ; θ = 30, 26.2%; θ = 50, 90.9%.
[33]	PCS ◦ θ Case1: θ = 30, 80.7%; θ = 50, 99.3% Case2: θ = 30, 73.0%; θ = 50, 99.4% Case3: θ = 30, 26.9%; θ = 50, 91.6% Case4: θ = 30, 15.0%; θ = 50, 80.3%
Wi-Mose [52]	P-MPJPE (mm); [51]: 37.6%; [52]: 29.7%.
DINN [54]	PCS ◦ θ 65.99% (50.27%) and 100% (99.82%).
Wi-Pose [51]	Basic Scenario; Occluded Scenario; Cross-domain Posture Construction.
Secure-Pose [88]	Cumulative Distribution Function (CDF); It has achieved a high detection accuracy of 95.1%.
Winect [77]	CDF (cm) Wi-Pose: 10.1 cm; Winect: 4.6 cm.
[55]	PCS ◦ θ PCS ◦ 50 Wi-Pose: 90.9%; [55]: 100.0%.
GoPose [97]	CDF (cm) GoPose: 4.7 cm

Here a is defined as the percentage of the length of the header in [49]; PCK@0.2 denotes that the trunk diameter is used as a reference in [48].

**Table 7:** Identification Results of LoS and NLoS.

System	Scenarios	Metric Performance
DINN [54]	Visible Scenario	PCS:100% (loosely), 66.5% (strictly)
	Through-wall Scenario	99.82% (loosely), 57.83% (strictly)
[33]	Visible Scenario	PCS ◦ θ same volunteer: θ = 25, 26.7%; θ = 30, 80.7%; θ = 40, 98.7%; θ = 50, 99.3% different volunteers: θ = 25, 2.5%; θ = 30, 26.9%; θ = 40, 77.2%; θ = 50, 91.6%
	Occluded Scenario	same volunteer: θ = 25, 23.4%; θ = 30, 73.0%; θ = 40, 98.9%; θ = 50, 99.4% different volunteers: θ = 25, 1.6%; θ = 30, 15.0%; θ = 40, 60.1%; θ = 50, 80.3%
Wi-Pose [51]	Visible Scenario	Average joint localization errors (mm): 95.3
	Occluded Scenario	Average joint localization errors (mm): 121.6
Wi-Mose [52]	Visible Scenario	(The larger the P-MPJPE is, the larger the error is) 42.6 mm
	Occluded Scenario	46.8 mm
Winect [77]	Visible Scenario	Average joint localization errors (mm): 4.3
	Occluded Scenario	Average joint localization errors (mm): 5.5
GoPose [97]	Visible Scenario	Average joint localization errors (mm): 4.7
	Occluded Scenario	Average joint localization errors (mm): 5.5
